# Urinary profiles of luteinizing hormone, estrogen and progestagen during the estrous and gestational periods in giant pandas (*Ailuropda melanoleuca*)

**DOI:** 10.1038/srep40749

**Published:** 2017-01-16

**Authors:** Kailai Cai, Shangmian Yie, Zhihe Zhang, Juan Wang, Zhigang Cai, Li Luo, Yuliang Liu, Hairui Wang, He Huang, Chengdong Wang, Xiangming Huang, Jingchao Lan, Rong Hou

**Affiliations:** 1 The Sichuan Key Laboratory of Conservation Biology on Endangered Wildlife, Chengdu Research Base of Giant Panda Breeding, Chengdu, Sichuan, People’s Republic of China

## Abstract

Luteinizing hormone (LH) is one of the main pituitary hormones that regulate ovulation, however its role has not been studied in giant panda. In this study, we developed an ELISA method for the detection of panda urinary LH. We analyzed urinary hormones of 24 female pandas during 36 breeding periods, we found females could easily be impregnated if the first mating occurred within 10 hours after LH peak. We also found the patterns of the ratios of urinary LH and progestagen in pandas that bred and successfully gave birth were significantly different from those that bred but failed to give birth. These data was the first to provide the urinary LH profiles during the estrous and gestational periods in pandas, and demonstrated that the appearance of the urinary LH peak indicated the timing of ovulation. The LH detection together with estrogen analysis makes the window for successful mating narrower than previously reported. Moreover, detection of urinary LH and progestagen can be used to discriminate between pregnancies and pseudopregnancies/miscarriages in the species. Thus, our findings suggest that LH not only plays a critical role in regulating ovulation but also plays an important role in maintaining pregnancy in the giant panda.

The giant panda (*Ailuropoda melanoleuca*) is one of the most endangered animals in the world. In the wild, the giant panda survives mainly in the mountains on the eastern edges of the Tibetan Plateau in the provinces of Sichuan, Shaanxi and Gansu in China^1^. As an endangered species, the significance of giant panda conservation goes far beyond protection efforts. This conservation generally consists of both *in-situ* projects and *ex-situ* efforts, with captive breeding being an important part of the *ex-situ* efforts^2^. Although captive breeding has produced many giant panda cubs, the number of cubs fit for breeding is still insufficient for creating and maintaining a genetically diverse population. Therefore, a need exists for continuous captive breeding research, which is crucial for giant panda *ex-situ* conservation efforts. However, scarce research resources have led to a lack of fundamental knowledge about the breeding process of giant pandas including the regulation of the reproductive endocrine system in the species.

Female giant pandas have a single annual estrous period that generally lasts for only a few days (24–72 h) between February and May each year^3^. Therefore, accurate monitoring of the estrous cycle to pinpoint the time of ovulation is a key aspect for giant panda breeding research because the timing of ovulation is essential for the success of timed mating or artificial insemination (AI). The analysis of urinary metabolites of estrogen and progestagen currently used as the main method to understand the estrous cycle of giant pandas^4^,^5^,^6^,^7^, however, this type of analysis alone has not been adequate in determining the precise timing of ovulation. The reported success rate of AI for giant pandas has only been 25% worldwide^8^ with the imprecise estimation of the timing of ovulation noted as the most likely cause of AI failure. As such, alternate methods of monitoring the estrous cycle to pinpoint the time of ovulation should prove to be critical for improving the AI success rate.

Luteinizing hormone (LH), one of the main pituitary hormones that regulates follicular development and ovulation in mammals^9^, has been used to determine the precise time of ovulation in several species^10^,^11^. Accordingly, the detection of LH may be an effective method to determine the time of ovulation in giant pandas. Unfortunately, no reliable detection method for LH in giant pandas exists at the moment. It has been reported that a canine serum LH immunochromatographic kit can be used on giant pandas^12^. However, this method was only a qualitative analysis and no exact variation of LH on giant panda estrus was presented.

Therefore, the development of a specific quantitative assay for giant panda LH could enhance our understanding of the giant panda reproductive process. In the present study, we first developed a specific ELISA for detecting giant panda LH in urine. Using this method, we then analyzed the variations of urinary LH during periods of estrus and pregnancy. Finally, we combined the LH results with changes in urinary estrogen and progestagen to explore the role that the determination of urinary LH can have in giant panda breeding.

## Results

### Validation of our LH ELISA

**Figure 1:**
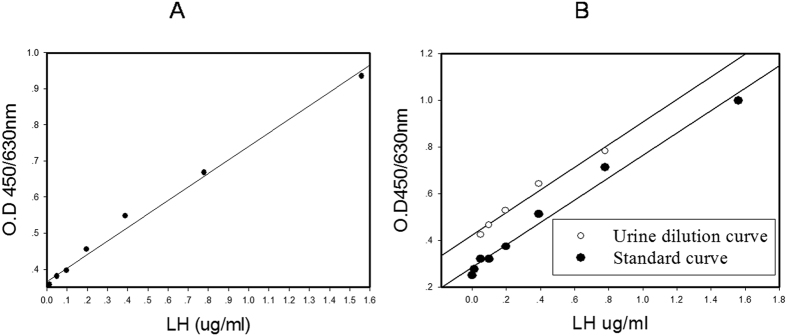
(**A**) LH ELISA standard linear regression curves. The concentration standards were 1.56 ug/mL, 0.78 ug/mL, 0.39 ug/mL, 0.195 ug/mL, 0.098 ug/mL, 0.049 ug/mL, 0.012 ug/mL and 0 ug/mL, the absorbance was spectrophotometrically read in a double wave length at 450 nm and 630 nm. (**B**) Parallel test curve with urine samples being double dilution to the test LH.

The standard curves of our LH ELISA were linear regression with 10 ng/mL of sensitivity (Fig. 1A). The serially diluted giant panda urine samples presented a dose response that was parallel to the standard curve (Fig. 1B). The intra- and inter-assay variations were 4.5 ± 0.93% and 2.16 ± 1.42% at 1.56 ng/ml (High level QC. sample), 3.5 ± 0.79 and 9.40 ± 1.66 at 0.09 ng/ml (Low level QC. Sample) (Table 1). The accuracy of the assay was 105.6 + 0.132% (Table 2) and the equation was y = 41.17 + 0.76x. These data demonstrate the effectiveness of our LH ELISA technique for detecting LH in giant panda urine.

### Urinary estrogen, progestagen and LH

**Figure 2:**
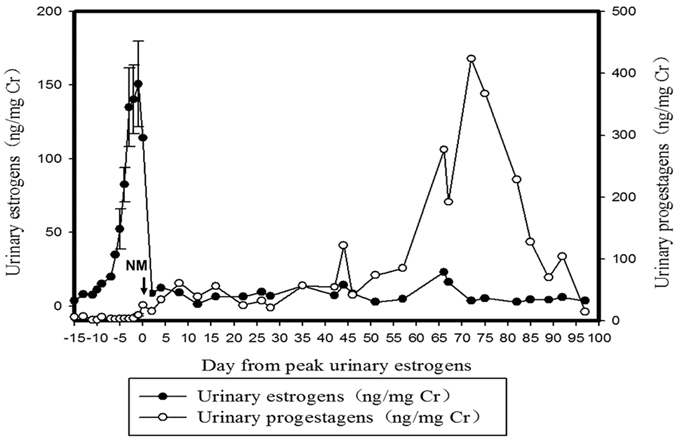
Typical estrogen and progestagen profiles during estrous and pregnancy periods. Data is aligned to the day of peak urinary estrogen, means ± SD estrogen profile in estrous period. Arrow indicate day of NM.

The typical profiles of urinary estrogen and progestagen concentrations are shown in Fig. 2. The urinary estrogen levels experienced a rapid increase from baseline to peak excretion over the course of 1–2 weeks, followed by a drastic decline at the presumptive time of ovulation, and then maintained at baseline levels throughout the pregnancy period. The urinary progestagen levels were at baseline during the period of rapid estrogen increase, followed by a gradual increase during the period of estrogen decline. Afterward, a rapid increase occurred during the pregnancy period and then dropped down again to the baseline after the female had given birth.

**Figure 3:**
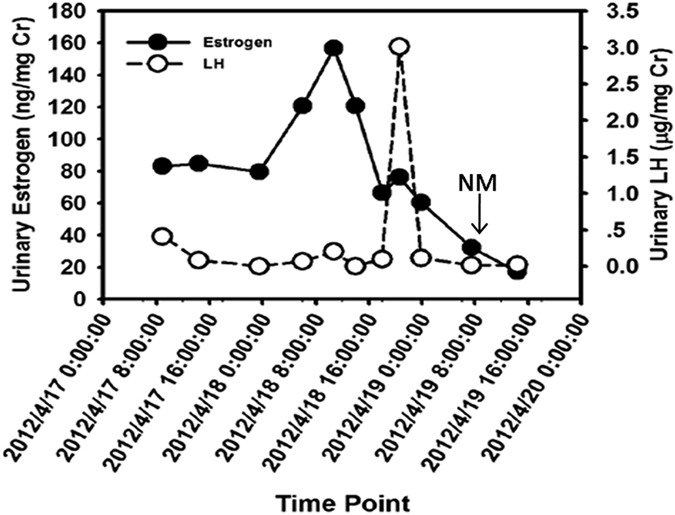
Typical change in estrous on LH and estrogen. Arrow indicate day of NM.

The urinary LH exhibited persistent volatility during the estrous period. However, there was a peak of LH after the estrogen peak. A typical change of urinary LH and estrogen in one estrus is shown in Fig. 3. All of the changes in urinary LH and estrogen during estrus periods are shown in Supplemental Figure 1. The interval time between LH peak and estrogen peak was different among individual giant pandas with the shortest interval time being 3 hours and 37 minutes. The interval time of estrogen peak and LH peak in the 24 giant pandas during the 36 estrous periods are shown in Table 3.

### Comparison of the interval time between the estrogen surge and LH surge during the estrous periods in female pandas that successfully gave birth and those that were bred yet did not give birth

During perioestrus, for both test groups, urinary estrogen profiles gradually rose from baseline to peak excretion over the course of 1–2 weeks, and then declined precipitously at the presumptive time of ovulation. Thereafter, both estrogen and LH peaked (Fig. 3). The average interval time between the estrogen peak and the LH peak in the successful birth group (11.2 ± 5.4 h) was shorter when compared to the group that did not give birth (14.8 ± 7.4 h), but the difference did not reach statistical significance. However, as shown in the Table 4, in the successful birth group, six female giant pandas had their first insemination (either through natural mating and/or AI) within 10 hours after the LH peak, eight females had their first insemination between 10 hours and 28 hours after the LH peak, and one female had its first insemination before the LH peak. By contrast, in the group that did not give birth, one female panda had its first insemination within 10 hours after the LH peak, 16 females had their first insemination between 10 hours and 28 hours after the LH peak, and four females had their first insemination time before the LH peak. These differences were statistically significant (*P* < 0.01; Table 4). The results here indicate that the observation of the interval time between the estrogen surge and LH surge during the estrous periods may be used to determine the mating times for female giant pandas.

### Comparison of patterns of progestagen and LH after mating between giant pandas that successfully gave birth and those that were bred yet did not give birth

**Figure 4:**
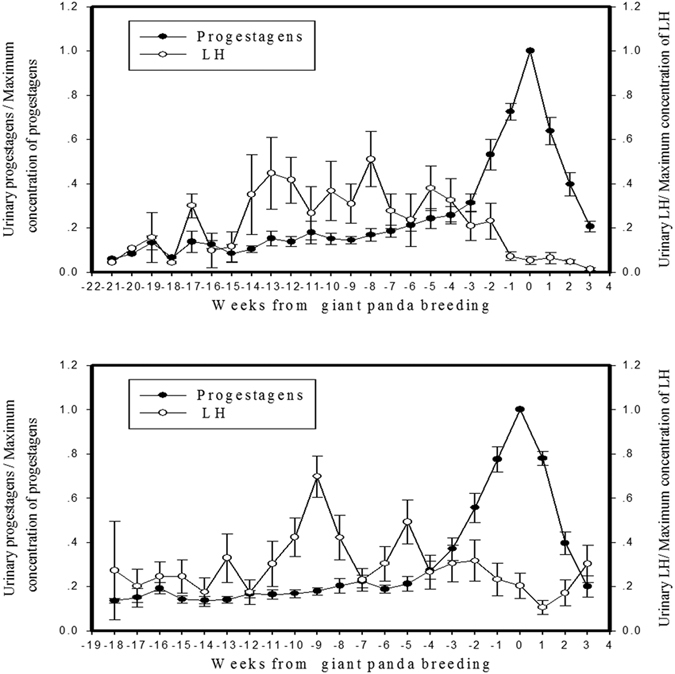
The pattern of progestagen and LH concentration after mating. Data is aligned to the week of peak urinary progestagen and presented as the means ± SEM. (**A**) Group with cubs, n = 14 (**B**) group without cubs, n = 20.

As shown in Fig. 2, the patterns of progestagen in both groups had only one peak during the pregnancy period. To compare whether there was a difference between the two groups, we presented the concentration of progestagen as a ratio of the concentration of progestagen to the maximum value of the progestagen for every week during the pregnancy period, and named the week of maximum progestagen concentration as week 0. Meanwhile, the concentration of LH was expressed as a ratio of the concentration of LH to the maximum value of the LH every week (Fig. 4). As shown in Fig. 4A, the ratios of progestagen and LH in the successful birth group showed the same pattern (i.e., the changes of LH were volatile with multiple peaks during the first rise of progestagen, followed by a decline to a low level during the second rise of progestagen and finally a drop down to the baseline). However, the ratios of progestagen and LH in the unsuccessful birth group showed that there was a further rise after LH went down to the baseline (Fig. 4B). Statistical analysis showed that there was a significant difference between the two groups (P = 0.001; Table 5). The results here indicate that it is possible to distinguish whether or not a mated female panda would give birth by observing the patterns of urinary LH changes.

## Discussion

For humans, pregnancy can be detected by using B-ultrasound and measurements of serum/urinary HCG. However, there are no reliable methods so far to determine whether giant pandas are pregnant, pseudo pregnant or have reabsorbed the fetus after AI or natural mating. In our study, all the subjects were inseminated successfully through AI or natural mating and all the pandas showed a similar pattern of progestagen profile during gestational periods. In addition, there were two giant pandas that gave birth following AI and two giant pandas that naturally mated and did not give birth, suggesting that breeding method was not a key factor in determining a successful birth. Therefore, we divided the animals into two groups: individuals that bred and successfully gave birth and individuals that were bred and did not give birth.

In the present study, we found that during the perioestrual interval, giant pandas experienced a rapid increase and then a decline in urinary estrogen metabolite concentrations. After the perioestrual interval, during a period of 1–2 weeks, the urinary estrogen metabolite concentrations rose gradually from baseline to peak excretion followed by a sudden decline at the presumptive time of ovulation. This pattern is very similar to patterns reported in a number of previous studies^3^,^13^,^14^.

For over 25 years the analysis of urinary estrogen metabolites has been used to estimate the time of ovulation in captive giant pandas^3^,^14^,^15^,^16^,^17^,^18^,^19^,^20^. Notorious for a short, annual estrous period, the ability to estimate or predict the timing of ovulation in giant pandas is instrumental for improving the natural mating and/or AI success rate, thus further reinforcing the *ex situ* population. As such, the measurement of urinary estrogen metabolites has been demonstrated to be a relatively effective method in determining the time of natural mating and/or AI^3^,^4^,^5^.

Animal managers at captive facilities often try to conduct natural mating or AI for giant pandas as soon as peak estrogen levels have been detected in urine samples and/or behavioral signs have been observed that suggest estrous has occurred. However, if the behavioral signs of a female giant panda are not clear, the timing of ovulation in the panda cannot be estimated by using urine tests alone. To compensate, the female giant panda is often subjected to two or more natural mating sessions and/or AI^4^,^5^,^13^. Each time that AI is performed, anesthesia is administered to the animal which could potentially endanger the health of the animal. Moreover, if pairing individuals for natural mating does not occur at the optimal time, then the risk of aggression between male and the female pandas can occur, risking harm to the animals and further increasing the rate of reproductive failure.

As described above, LH is one of the main pituitary hormones to regulate follicular development and ovulation^9^, and the measurement of LH has been used as a main method to determine the timing of ovulation^10^,^11^. In humans, ovulation occurs 28–36 hours after the beginning of blood LH rise or 8–20 hours after the peak in LH^21^. Although the ovulation time in giant pandas is unknown, the measurement of changes in urinary LH during the estrous period may enhance our understanding of their reproductive process.

As giant pandas are an endangered species, noninvasive hormone analysis, such as urine or fecal analysis, offer the best way to study the reproductive process of giant pandas. For humans, it has been reported that the peak LH level in plasma and urine usually occur on the same day^22^. Considering the structure of LH is highly similar across mammalian species, this pattern may be the case in giant pandas as well^23^,^24^. Given the highly similar structure of canine and giant panda LH, it had been reported that a commercial canine serum LH assay kit was used with panda serum, in which either a long LH surge or a slow LH clearance rate in most estrous cycles was found^12^. However, the canine serum LH assay kit was only a qualitative analysis with a small sample size (n = 4), and the authors did not analyze the relationship between LH and ovulation.

In our study, we developed the antibodies against a giant panda LH beta subunit, and identified the specificity of the antibodies by Western blot. By using the specific antibodies, we established a quantitative double antibody sandwich ELISA for urinary LH measurement in giant pandas. The accuracy, repeatability and sensitivity of the ELISA were demonstrated by a number of standard ELISA development approaches such as a parallel test, recovery test, and variation analysis.

By using our novel method, we analyzed 36 estrous urine samples of 24 female giant pandas and found that urinary LH peaks always appeared after urinary estrogen peaks although the interval time between the LH peak and estrogen peak were different among individual giant pandas. Interestingly, the first insemination in most giant pandas that gave birth occurred within 10 hours or between 10 hours and 28 hours after the LH peak. This result suggests that the appearance of a urinary LH peak may indicate that the timing of ovulation in giant pandas is similar to the timing of ovulation in humans, which occurs 8–20 hours after the blood LH peak^21^. This in turn suggests that for breeding practices, the LH variation analysis together with estrogen variation analysis in giant panda breeding may make the window for successful mating narrower than previously appreciated^4^,^5^, however, further studies are necessary to fully confirm this hypothesis.

In the present study, we also observed that the urinary progestagen levels of the female pandas’ experienced a baseline during the period of rapid estrogen increase, followed by a gradual increase during the period of estrogen decline. Afterward, a rapid increase occurred during the gestation period before dropping down to the baseline again. This excretion pattern of urinary progestagen is very similar to the patterns reported in a previous study^3^. In humans, early on in pregnancy just after conception has taken place, a systemic rise of progestagen level is produced by the corpus luteum in the ovary followed by a second rise of progestagen levels produced by the placenta trophoblast cells during late pregnancy. This unique bi-phase pattern has been demonstrated in giant pandas in the present study and other urinary hormone studies^3^ as well as in fecal hormone monitoring studies^25^.

Because the progestagen excretion pattern throughout this period was very similar between real pregnancies and pseudopregnancies or miscarriages we could not use progestagen to discriminate between these situations^25^. Nevertheless, in our study, we found that the ratios of progestagen and LH dropped down to a baseline level just before parturition in the group that successfully birthed while the ratios in the group that did not had a further rise. Thus, it may be speculated that the second peak of urinary progestagen in miscarriages or pseudopregnancies in animals came from the corpus luteum rather than the placenta. In such cases, LH may play a continuous stimulation role in the corpus luteum cells to produce the progestagen. When progestagen levels drop down, a negative feedback between the progestagen and LH may raise the LH levels again. In contrast to pseudopregnancies or miscarriages, progestagen in successful pregnancies for giant pandas may be secreted by both the corpus luteum and placenta, but mainly the placenta. Just before parturition, progestagen levels drop down and the cub is born. The relationship between placental gonadotrophins and progestagen ends, and as a result, no further rise in LH is observed.

It is well established that the major gonadotrophin secreted by the placenta is chorionic gonadotropin (CG) in mammals, including humans, with placenta-dependent pregnancies. Unfortunately, there is no effective method to measure the hormone in the urine of giant pandas yet. Therefore, to verify our speculation, novel detection tools for giant panda CG or other relative placental hormones needs to be developed for the future studies.

In summary, in the present study, we have developed a specific ELISA for the detection of giant panda LH in urine for the first time. By using this method, we have analyzed variations of urinary LH in thousands of samples from a relatively large number of female giant pandas during two estrous and gestational periods. We also statistically compared the results between female pandas that successfully birthed and those that did not, which previous studies have not performed. Interestingly, we found that the appearance of urinary LH peak during estrous may be correlated with the timing of ovulation in giant pandas. The combination of urinary estrogen peak with LH peak may be more accurate in determining the time of natural mating and/or AI than using urinary estrogen tests alone. This in turn will increase the success rate of giant panda breeding. In our study, we also found that there was a different pattern in urinary LH and progestagen during the gestation period between giant pandas that successfully gave birth and those that did not. This may be used to distinguish real pregnancies from pseudopregnancies or miscarriage in the species. However, the underlying mechanism surrounding these findings still needs to be investigated in future studies.

## Materials and Methods

### Study animals and urinary samples

Giant pandas were housed at the Chengdu Research Base of Giant Panda Breeding in Sichuan Province, People’s Republic of China. Twenty-four captive, sexually mature female giant pandas were investigated in this study. Of the 24 giant pandas, 17 took part in the breeding program of 2012, in which seven cubs were produced (one set of twins and five single births). Nineteen giant pandas, including 12 females that were part of the breeding program in 2012 (six that had successful births and six that did not) took part in the breeding program of 2013 as well, in which 14 cubs (five sets of twins and four single births) were produced. A total of 21 cubs in 15 pregnancies (six sets of twins, 11 single births) were born in the two breeding years (Table 6). In order to analyze the relationship between the estrogen surge and LH surge during the estrous periods and progestagen and LH during pregnancy, we divided the females into two groups: one that successfully gave birth to cubs and one that did not. Both groups were inseminated via natural breeding and/or AI (Table 6). This research complied with the legal requirements of the government of China and the safety and ethical protocols of the China Wildlife Conservation Association’s Principles for the Ethical Treatment of Endangered Species. The methods used in this study were assessed and approved by directors and appropriate staff of the Research, Animal Disease Prevention and Control Department (Veterinary), Animal Husbandry, and Management departments of the Chengdu Research Base of Giant Panda Breeding, and by the Chinese Association of Zoological Gardens, before being initiated. In addition, oversight of the breeding, care, and research activities at the Chengdu Research Base is performed by the State Forestry Administration of the People’s Republic of China.

Urinary samples from 36 estrus and pregnancy periods were collected. The sample collection process was designed to be as follow: (i) one urinary sample per panda per day during estrus period, (ii) one sample per every four hours at the peak phase of the estrous period, (iii) three samples per week after the panda mated, and (iv) one sample per day during the late stage of pregnancy. The urinary samples were aspirated from the enclosure floor with clean plastic syringes and then transferred to a 12–75 mm plastic tube, labeled with an animal identification number and a date of collection. All the samples were stored at −20 °C until analysis.

### Design and synthesize of special peptides for giant panda LH

LH, like other anterior pituitary hormones such as follicle stimulating hormone (FSH) and thyroid stimulating hormone (TSH), is composed of two un-identical subunits, alpha and beta. The alpha subunit is identical for all of these hormones while the beta subunit is distinct in each. Therefore, for the immunological detection of these hormones, specific antibodies against the respective beta subunit should be prepared.

Commercially, an amino acid sequence of RPWCHPINAILAVEKEGCPV from N-Term human LH beta subunits and an amino acid sequence of CGGPKDHPLTCDHPQLSGLL from C-Term human LH beta subunits have been used as immunogens to develop special antibodies against human LH. These amino acid sequences are located at the LH beta subunit antigenic determinant. Because the amino acid sequence length of the giant panda LH beta subunit is the same as a human’s with a 76% homology between the two species, we designed two special peptides derived from a unique region of the giant panda LH beta subunit: one corresponding to the C-Term at the same location as in the human LH beta subunit and another one corresponding to the N-Term at the same location as in the human LH beta subunit. The peptides, identified by the letter code of RPLCRPINATLAAENEAC (N-Term) and CGGPRAQPLACDRPPLPGLL (C-Term), were synthesized and purified by a high-performance liquid chromatography (HPLC) from Sangon Biotech (Shanghai) Co., Ltd., and named LH-N and LH-C, respectively. The resulting peptide sequences showed some amino acid variations when compared to other vertebrate species (see Supplemental Table S1), although LH beta subunit is highly conserved in vertebrates.

### Preparation of specific antibodies against giant panda LH beta subunit

The two synthesized peptides were conjugated with KLH as immunogens by Sangon Biotech (Shanghai) Co., Ltd., and named LH-N-KLH and LH-C-KLH, respectively. Six female New Zealand white rabbits divided into two groups were subcutaneously immunized with LH-N-KLH or LH-C-KLH at multiple sites on their back (Approval ID: SCXK (Sichuan) 2013–24). The initial injection was carried out with 0.5 mg of the conjugates mixed with complete Freund’s adjuvant (Sigma, USA). Next, subsequent boost injections were administered once a week for 4 weeks with incomplete Freund’s adjuvant (Sigma, USA) as an emulsifying agent. Three days after the final boost, six rabbits were euthanized by exsanguinations and whole blood samples from the rabbits were coagulated overnight at 4 °C, centrifuged to separate the serum at 4 000 × g for 10 min. Finally, titers of antibodies were determined by using an indirect enzyme-linked immunosorbent analysis (ELISA) with the synthesized special peptides of LH-N or LH-C. Positive serum containing anti-LH beta subunit antibodies and negative serum collected before immunization were diluted (from 1:500 to 1:40000) and their reactivity with LH-N or LH-C are shown in Supplemental Table S2. The anti-serums were stored at −80 °C until use.

### Identification, purification and labeling of the antibodies

The specificity of the antibodies was analyzed by using Western blot analysis with recombinant protein of panda LH beta subunit. The methods for the preparation of the recombinant protein are shown in Supplemental 1 and the results are shown in Supplemental Figures 2 and 3. As shown in Supplemental Figure 4, the antibodies specifically recognized the recombinant protein with a single band on the Western blot.

The antibodies were purified by using a protein G agarose (Pierce, USA) affinity chromatography in accordance with the manufacturer’s instructions. The purified antibodies were then concentrated by a 15 mL ultrafiltration centrifugal filter (Millipore) with the final concentrations being determined by a BCA-assay kit (Pierce, USA).

To biotin label the antibodies, buffer salts and small contaminants in the purified antibodies were first removed by using a dialysis cassette (Slide-A-Lyzer Dialysis Cassettes, 10 K MWCO, Pierce). The resulting purified antibodies were then labeled with biotin using an EZ-Link Sulfo-NHS-LC-Biotinylation Kit (Pierce, USA) in accordance with the manufacturer’s instructions.

### Development of a double antibody sandwich ELISA for detecting LH in giant panda urine

Briefly, the coated antibodies were diluted in 10 μg/mL carbonate buffer and 50 μL per well was added to 96-well ELISA plates. The plates were incubated overnight at 4 °C, washed with a washing buffer (PBST) and then blocked with 300 μL per well blocking buffer (2% BSA) for overnight at 4 °C. Afterward, 50 μL per well of 6 labeled serum antibodies dilutions and three giant panda urine samples were added in the antibody coated antibodies plates and incubated at 37 °C for 30 min. After washing 3 times, 50 μL per well of streptavidin-HRP conjugate (1:300 dilution, Pierce) was added and incubated at 37 °C for 30 min. Finally, 100 μL of substrate chromogen (TMB, Sigma) working solution was added and incubated for 15 min, and the enzymatic reaction was stopped with sulfuric acid (2 mol/L, 100 μL/well). The plates were read in a double wave length at 450 nm and 630 nm (Multiskan MK3, Thermo).

By using the method described above, a pair consisting of the best coated antibody (LH-N-3) and the best biotin labeled antibody (LH-C-2) was selected from 6 antibody preparations (Supplemental Table S3), and 1:4000 was considered the best dilution for the labeled antibody (Supplemental Table S4). Next, we used a synthesized peptide (RPLCRPINATLAAENEACCGGPRAQPLACDRPPLPGLL) from N-Term to C-Term of the giant panda LH beta subunit as a standard for this ELISA assay, which had a similar result and easier to obtain than recombinant protein. The standard was serially diluted with PBS from 1.56 μg/mL to 0 μg/mL to set up a standard curve.

Finally, to calculate the assay sensitivity we used 10 wells of 0 μg/mL standard, and repeated the experiment 10 times to calculate the variation coefficients within and between batches of the assay. We performed a parallel experiment with a serially diluted giant panda urine pool to compare with the standard curves and, in addition, a recovery experiment was carried out to evaluate the accuracy of the assay.

Based on the experiments described above, a double antibody sandwich ELISA for detecting urinary LH in giant pandas was developed. The LH concentrations in the panda urinary samples were determined by comparing with the standard curve. Further, the LH concentrations were indexed by creatinine (Cr) in order to adjust for variability in the urine dilution^26^ and expressed as mass/mg Cr.

### Urinary estrogen and progestagen assays

A single antibody estrogen glucuronide EIA (R583; C. Munro, University of California, Davis, CA was used to quantify estrogen concentrations in urinary samples^27^,^28^. The inter-assay CV for two internal controls (n = 10 assays) was 13.5% and the intra-assay CV was <10%. Conversely, a single antibody P4 EIA (CL425; C. Munro^29^) was used to quantify progestagen concentrations in urinary samples. The inter-assay CV for the two internal controls (n = 54 assays) was 12.2% and the intra-assay CV was <10%. All hormone concentrations were indexed by creatinine (Cr) in order to adjust for variability in the urine dilution^26^ and expressed as mass/mg Cr.

### Statistical analysis

Statistical analysis was performed using the SPSS software package (14th edition, Abacus Concepts, Berkeley, CA, USA). The Pearson Chi-square test was used to compare the frequency differences in interval time of the estrogen surge and LH surge during the estrous periods and the patterns of progestagen/LH after mating between giant pandas with and without cubs. P < 0.05 was considered to indicate a statistically significant difference.

## Additional Information

**How to cite this article**: Cai, K. *et al*. Urinary profiles of luteinizing hormone, estrogen and progestagen during the estrous and gestational periods in giant pandas (*Ailuropda melanoleuca*). *Sci. Rep.* **7**, 40749; doi: 10.1038/srep40749 (2017).

**Publisher's note:** Springer Nature remains neutral with regard to jurisdictional claims in published maps and institutional affiliations.

## Supplementary Material

Supplementary Information

## Figures and Tables

**Table 1:** The intra- and inter-assay variations of LH ELISA.

	Inter-assay CV% (n = 10)	Intra- assay CV% (n = 8)
High level QC. sample	2.16 ± 1.42 (1.56 ng/ml)	4.5 ± 0.93 (1.56 ng/ml)
Low level QC. sample	9.40 ± 1.66 (0.09 ng/ml)	3.5 ± 0.79 (0.09 ng/ml)

Values presented by means ± SE.

**Table 2:** The recovery rate of LH ELISA for giant panda.

Urine	Urine + 100 ng (ng/ml)			Urine + 200 ng (ng/ml)			Urine + 400 ng (ng/ml)			
Actual value	Actual value	Theoretical value	Recover rate	Actual value	Theoretical value	Recovery rate	Actual value	Theoretical value	Recovery rate	The mean recover rate
20	117	120	0.983 ± 0.121	222	220	0.995 ± 0.015	497	420	1.191 ± 0.117	1.056 ± 0.132
14	101	114		210	214		443	414		
20	131	120		219	220		543	420		

Data are presented as means ± SD.

**Table 3:** Summary of studied giant pandas information about the time of the mating, estrogen peak and LH peak.

Female studbook	The time of estrogens peak (yyyy/m/d h:m)	Mating time (yyyy/m/d h:m)	The time of LH peak (yyyy/m/d h:m)	The time between LH peak and estrogens peak (d h:m)	The time between LH peak and mating time (d h:m)
425	2012/3/12 16:10	2012/3/13 14:05	2012/3/13 7:30	0 15:20	0 6:35
425	2013/4/24 7:13	2013/4/25 7:37	2013/4/24 10:50	0 3:37	0 20:47
593	2012/3/26 20:00	2012/3/27 18:00	2012/3/27 9:50	0 13:50	0 8:10
387	2012/4/3 13:20	2012/4/4 10:59	2012/4/3 21:25	0 8:05	0 13:34
523	2012/4/12 14:20	2012/4/13 21:37	2012/4/13 7:20	0 17:00	0 14:17
635	2012/4/15 7:50	2012/4/16 14:04	2012/4/15 23:40	0 15:50	0 14:24
561	2012/4/18 10:45	2012/4/19 9:38	2012/4/18 20:40	0 9:55	0 12:58
522	2013/3/19 23:55	2013/3/20 15:17	2013/3/20 13:45	0 13:50	0 1:32
401	2013/4/4 0:50	2013/4/4 18:57	2013/4/4 14:10	0 13:20	0 4:47
491	2013/4/11 3:40	2013/4/12 0:40	2013/4/11 19:00	0 15:20	0 5:40
680	2013/4/13 23:27	2013/4/14 18:40	2013/4/14 3:36	0 4:09	0 15:04
537	2013/4/18 7:40	2013/4/19 7:50	2013/4/18 14:10	0 6:30	0 17:40
645	2013/4/18 14:30	2013/4/19 3:30	2013/4/19 9:20	0 18:50	*
681	2013/4/30 7:00	2013/5/1 14:45	2013/4/30 10:40	0 3:40	1 4:05
652	2013/5/7 22:00	2013/5/8 16:00	2013/5/8 13:00	0 15:00	0 3:00
480	2012/3/18 16:30	2012/3/20 0:47	2012/3/19 6:10	0 13:40:	0 18:37
480	2013/3/1 7:06	2013/3/2 10:12	2013/3/1 21:40	0 14:34	0 12:32
598	2013/5/11 7:30	2013/5/12 3:13	2013/5/11 15:30	0 8:00	0 11:43
598	2012/3/29 11:00	2012/3/30 22:10	2012/3/29 22:40	0 11:40	0 23:30
765	2012/4/1 19:10	2012/4/2 22:50	2012/4/2 7:50	0 12:40	0 15:00
765	2013/3/18 4:13	2013/3/19 14:14	2013/3/18 7:45	0 3:32	1 6:29
554	2012/4/1 20:00	2012/4/3 10:07	2012/4/2 22:10	1 2:10	0 11:57
554	2013/4/22 3:50	2013/4/23 7:12	2013/4/22 16:05	0 12:15	0 15:07
407	2012/4/18 6:30	2012/4/19 9:15	2012/4/18 19:05	0 12:35	0 14:10
407	2013/3/25 20:05	2013/3/26 12:00	2013/3/26 21:30	1 1:25	*
637	2013/4/10 3:40	2013/4/11 0:20	2013/4/10 7:35	0 3:55	0 16:45
637	2012/4/18 5:45	2012/4/19 11:22	2012/4/18 8:10	0 2:25	1 3:12
645	2012/4/22 7:40	2012/4/23 14:52	2012/4/22 21:05	0 13:25	0 17:47
522	2012/5/15 0:13	2012/5/16 18:17	2012/5/15 19:30	0 19:17	0 22:47
681	2012/5/13 14:30	2012/5/14 23:28	2012/5/14 8:00	0 17:30	0 15:28
678	2013/3/119:00	2013/3/2 20:48	2013/3/2 22:25	1 3:25:00	*
665	2013/3/2 19:05	2013/3/3 20:10	2013/3/3 21:40	1 2:35:00	*
671	2013/4/3 19:45	2013/4/4 18:59	2013/4/4 11:30	0 15:45	0 7:29
453	2013/4/15 17:00	2013/4/16 17:54	2013/4/16 3:50	0 10:50	0 14:04
537	2012/3/25 20:30	2012/3/27 6:15	2012/3/26 14:40	0 18:10	0 15:35
652	2012/3/20 19:09	2012/3/21 19:57	2012/3/23 9:00	2 13:51	*

^*^The mating time was before the peak of LH.

**Table 4:** Statistical analysis the relationship between the mating time and LH peak time with pregnancy success rates, P = 0.027 < 0.05 by spss.

Interval of first mating and LH peak after estrogen peak (h)	successful births	unsuccessful births	birth rate (%)	P value
Less than 10 hours	6	1	85.71%	0.027
Between 10 h and 28 h	8	16	33.33%	
Before LH peak	1	4	25.00%	

**Table 5:** Statistical analysis the relationship between the ratio date of progestagens and LH profile pattern with pregnancy success rates, P = 0.001 < 0.01 by spss.

Ratio date of progestagens and LH profile pattern	Successful births	Unsuccessful births	P value
Pattern A	14	9	0.001
Pattern B	0	11	

**Table 6:** Summary of studied giant panda females at Chengdu Research Base of Giant Panda Breeding, Chengdu, Sichuan, People’s Republic of China.

Female studbook	Age (yr)	Breeding type	First Mating date	Date of birth	No. of cubs
425	17	NM	Mar.13, 2012	Sep.14, 2012	1
	18	NM	Apr.25, 2013	Aug.19, 2013	1
480	13	AI	Mar.20, 2012		None
	14	AI	Mar.2, 2013		None
652	6	AI	Mar.21, 2012		None
	7	NM	May.8, 2013	Aug.22, 2013	2
537	11	AI	Mar.27, 2012		None
	12	NM	Apr.19, 2013	Aug.14, 2013	1
593	8	NM	Mar.27, 2012	Aug.19, 2012	1
598	7	AI	Mar.30, 2012		None
	8	AI	May.12, 2013		None
765	6	AI	Apr.2, 2012		None
	7	AI	Mar.19, 2013		None
554	10	AI	Apr.3, 2012		None
	11	AI	Apr.23, 2013		None
387	20	NM	Apr.4, 2012	Jul.28, 2012	1
523	12	AI	Apr.13, 2012	Sep.12, 2012	2
635	6	AI	Apr.16, 2012	Aug.12, 2012	1
407	18	AI	Apr.19, 2012		None
	19	AI	Mar.26, 2013		None
561	9	NM	Apr.19, 2012	Aug.25, 2012	1
637	6	AI	Apr.19, 2012		None
	7	AI	Apr.11, 2013		None
645	6	AI	Apr.23, 2012		None
	7	NM	Apr.19, 2013	Aug.6, 2013	2
681	5	AI	Mar.14, 2012		None
	6	NM	Mar.1, 2013	Aug.27, 2013	2
522	12	AI	May.16, 2012		None
	13	NM	Mar.20, 2013	Jul.23, 2013	2
678	6	NM	Mar.2, 2013		None
665	6	AI	Mar.3, 2013		None
671	6	AI	Apr.4, 2013		None
401	20	NM	Apr.4, 2013	Jul.10, 2013	1
491	14	NM	Apr.12, 2013	Jul.17, 2013	1
680	6	NM	Apr.14, 2013	Aug.23, 2013	2
453	16	NM	Apr.16, 2013		None
