# Supplementary material for: Urinary profiles of luteinizing hormone, estrogen and progestagen during the estrous and gestational periods in giant pandas (Ailuropda melanoleuca)
Source: Sci Rep. 2017 Jan 16;7:40749. doi: 10.1038/srep40749 (PMC5238505; doi:10.1038/srep40749)

Title: Urinary profiles of luteinizing hormone, estrogen and progestagen during the estrous and gestational periods in giant pandas (Ailuropda melanoleuca)

Authors: Kailai Cai, ShangmianYie, Zhihe Zhang, Juan Wang, Zhigang Cai, Li Luo, Yuliang Liu, Hairui Wang, He Huang, Chengdong Wang, Xiangming Huang, Jingchao Lan, Rong Hou*

Preparation of recombinant proteins panda LH beta

The complete coding sequence of the giant panda LH beta (*LHB*, NM_001304859.1) was derived from the GenBank database. First, 378 bases LHB gene fragment for the expression of the corresponding protein was synthesized, which contained LHB gene sequence from position 61 to position 426 and inserted Nde I restriction site at 5'starting end as well as XhoI restriction site at 3' end. The synthesized fragment was forward constructed to PET28b (Novagen, USA) vector and named LHB recombinant plasmid. The plasmid was identified by sequencing and restriction analysis (Supplemental Figure 2). Next, the LHB recombinant plasmid was transformed into the E.coli BL21 (DE3) strain (Novagen, USA) and used for induction by adding IPTG at an OD600 of 0.2 mM, and further cultured for 5 h at 37 ℃ using the empty vector transformed BL21 (DE3) as control. Afterward, the LHB recombinant plasmid was separated by sodium dodecyl sulfate-polyacrylamide gel electrophoresis (SDS-PAGE) (Supplemental Figure 3A). The LHB recombinant bacteria were harvested by centrifugation at 4000 g for 10 min, and the pellet was suspended in 20 mM Tris-HCl buffer, pH 8.0. The cell suspension was subjected to sonication with a 3-s burst and 10-s pause for 20 min in a cooling bath. The lysate was clarified by centrifugation at 10,000 g for 15 min at 4 ℃, and the supernatant was collected. For purification, the LHB recombinant protein was purified using affinity chromatography by Ni-NTA Agarose (Qiagen). The purified protein was identified

by SDS-PAGE (Supplemental Figure 3B) and the protein concentration was detected by a non-interference protein assay kit (SK3071, Shanghai Sangon Biotech). The results indicate that the recombinant protein bands were successfully induced by IPTG with a single protein band being displayed on the gel after purification.

Supplemental figure legends

Supplement Figure 1 LH and estrogen profiles during estrous. All the pictures are named with giant panda studbook and breeding year.

Supplemental Figure 2. Identification of recombinant plasmids (A) Sequencing of the LHB recombinant plasmid. (B) Restriction mapping analysis of recombinant plasmid

Supplemental Figure 3. Identification of recombinant protein (A) LHB recombinant protein profile by SDS-PAGE (B) LHB recombinant purificated protein by using affinity chromatography by Ni-NTA Agarose and identified by SDS-PAGE

Supplemental Figure 4 Antibodies specific detection (A) anti-LH beta subunit polyclonal antibody (LH-N) Western blot detection with LHB recombinant purificated protein (B) anti-LH beta subunit polyclonal antibody (LH-C) Western blot detection with LHB recombinant purificated protein. (C) Western Blot decetion with LHB recombinant purificated protein (original picture)

Supplemental tables

Supplemental Table S1. Unique amino acids sequences in LH beta subunit in various vertebrates

Animal vertebrate sources	Partial amino acid sequences (N-Term)	Partial amino acid sequences (C-Term)
Ailuropoda melanoleuca (giant panda)	RPLCRPINATLAAENEAC	CGGPRAQPLACDRPPLPGLL
Homo sapiens (human)	RPWCHPINAILAVEKEGC	CGGPKDHPLTCDHPQLSGLL
Mus musculus (mouse)	RPLCRPVNATLAAENEFC	CGGPRTQPMACDLPHLPGLL
Bos taurus (cattle)	RPLCQPINATLAAEKEAC	CGGPRTQPLACDHPPLPDIL
Equus caballus (horse)	RPLCRPINATLAAEKEAC	CGVFRDQPLACAPQASSSSD
Ovis aries (sheep)	RPLCQPINATLAAEKEAC	CGGPRTQPLACDHPPLPDIL

Supplemental Table S2 Detection of antibody titer in serum by indirect ELISA

Rabbit No.	Antigen	Blank	Negative				Positive			
			1:500	1:5000	1:20000	1:40000	1:500	1:5000	1:20000	1:40000
1	LH-N-KLH	0.127	0.28	0.141	0.14	0.131	2.13	1.99	1.695	1.443
2	LH-N-KLH	0.131	0.349	0.168	0.136	0.135	1.859	1.697	1.169	0.578
3	LH-N-KLH	0.129	1.317	0.252	0.15	0.151	2.054	1.824	1.535	1.218
4	LH-C-KLH	0.138	0.401	0.164	0.185	0.118	1.667	1.62	1.496	1.327
5	LH-C-KLH	0.174	0.387	0.152	0.159	0.123	1.604	1.598	1.473	1.339
6	LH-C-KLH	0.192	1.076	0.315	0.16	0.172	1.622	1.77	1.415	0.999

Supplemental Table S3. Coated antibody and labeled antibody pair selection

Coated antibody	Labeled antibody	Blank1	Blank2	Urine1	Urine2	Urine3
LH-N-1	LH-C-1	0.596	0.636	1.148	0.801	0.70
LH-N-1	LH-C-2	0.386	0.358	1.001	0.699	0.659
LH-N-1	LH-C-3	0.674	0.646	1.300	0.872	0.846
LH-N-2	LH-C-1	0.632	0.614	0.900	1.096	0.667
LH-N-2	LH-C-2	0.397	0.409	1.656	1.503	1.205
LH-N-2	LH-C-3	0.568	0.548	1.191	1.127	0.673
LH-N-3	LH-C-1	0.550	0.551	0.951	1.084	0.671
LH-N-3	LH-C-2	0.337	0.331	1.433	1.280	1.008
LH-N-3	LH-C-3	0.477	0.511	1.162	1.032	0.614
LH-C-1	LH-N-1	0.742	0.786	1.211	1.018	0.829
LH-C-1	LH-N-1	0.986	1.043	1.251	1.269	1.137
LH-C-1	LH-N-1	0.868	0.931	1.344	1.499	0.976
LH-C-2	LH-N-2	0.465	0.45	1.231	0.906	0.684
LH-C-2	LH-N -2	0.728	0.724	1.471	1.475	1.037
LH-C-2	LH-N -2	0.542	0.569	1.631	1.598	1.153
LH-C-3	LH-N -3	0.444	0.416	1.043	0.680	0.681
LH-C-3	LH-N -3	0.763	0.713	1.12	0.866	0.920
LH-C-3	LH-N -3	0.624	0.582	1.411	0.857	0.891

Supplemental Table S4. Working concentration of the labeled antibody

Coated antibody	Labeled antibody (LH-N-3)					
LH-N-3(10ug/mL)	dilution	1:1000	1:2000	1:3000	1:4000	negative
	O.D. value	0.301	0.192	0.158	0.148	0.143

Supplemental Figures

Supplemental Figure1

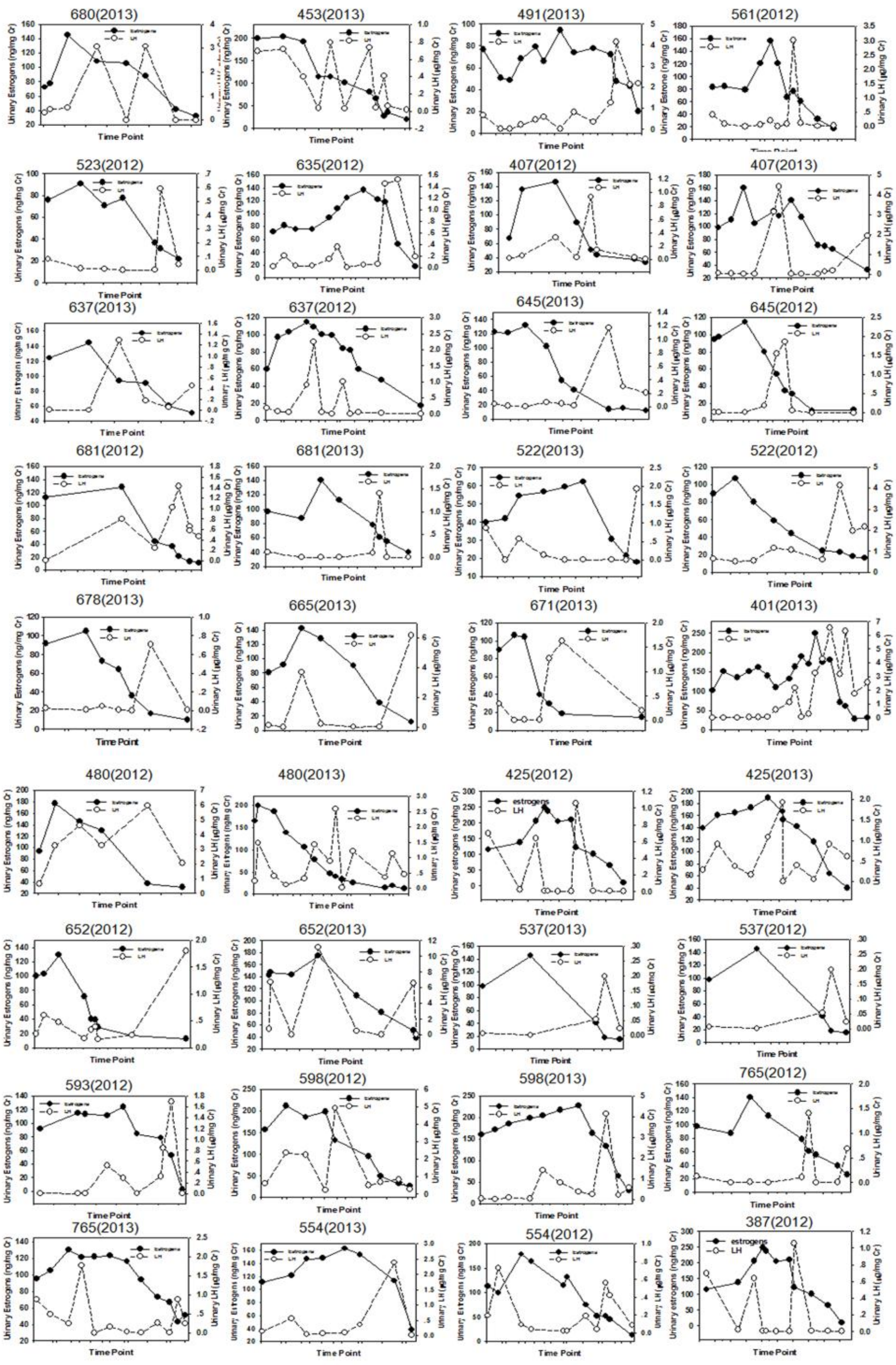

Supplemental Figure2

A

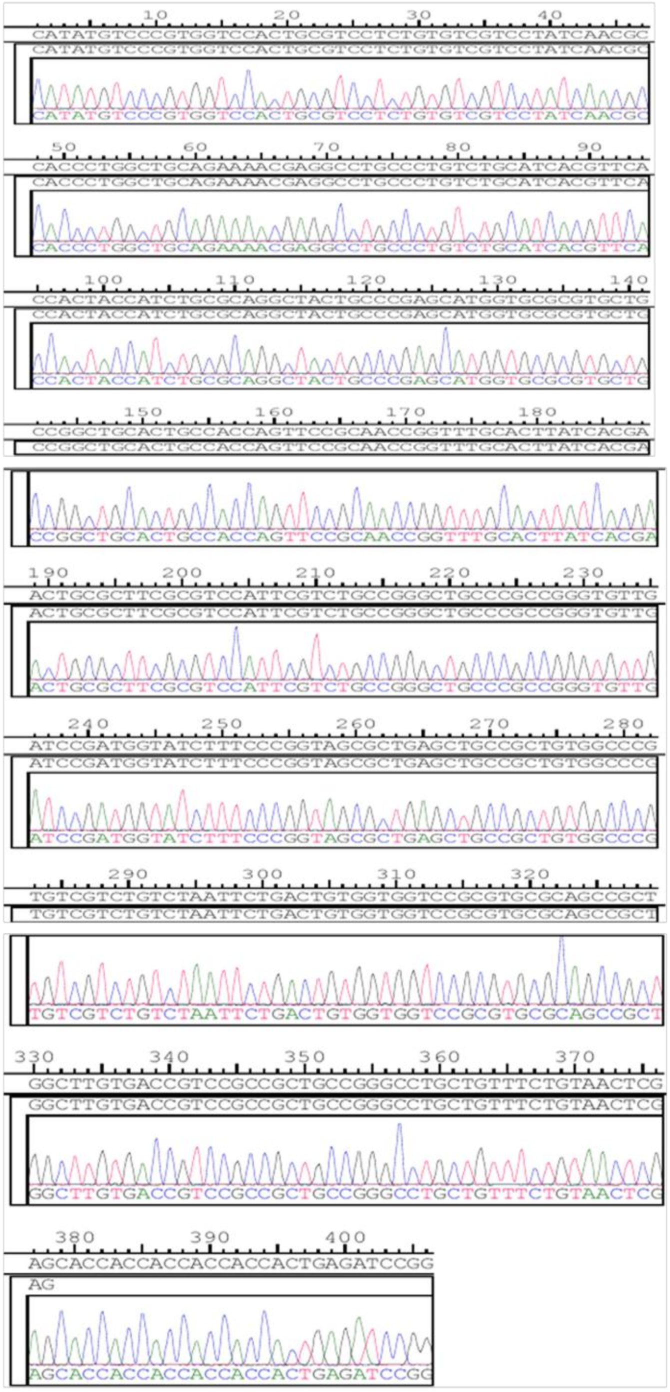

B

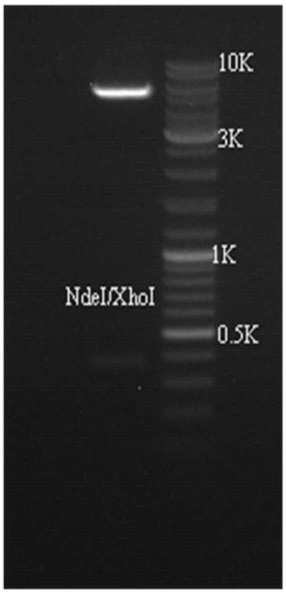

Supplemental Figure3

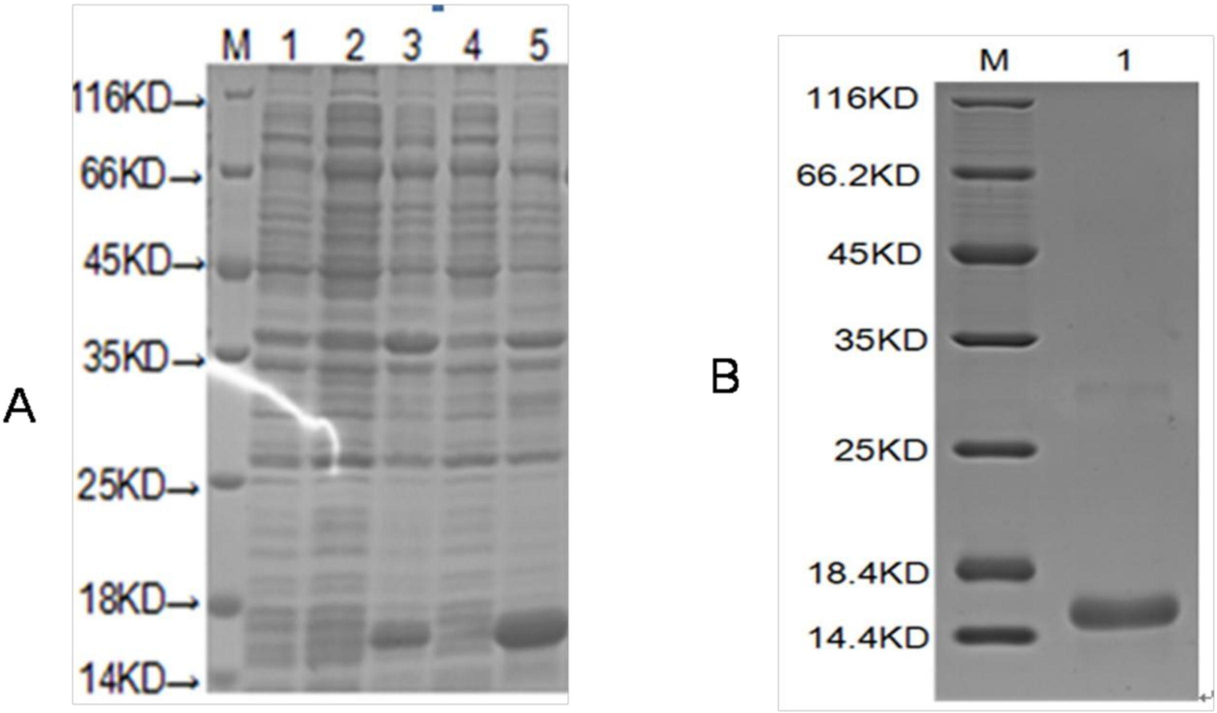

Supplemental Figure 4

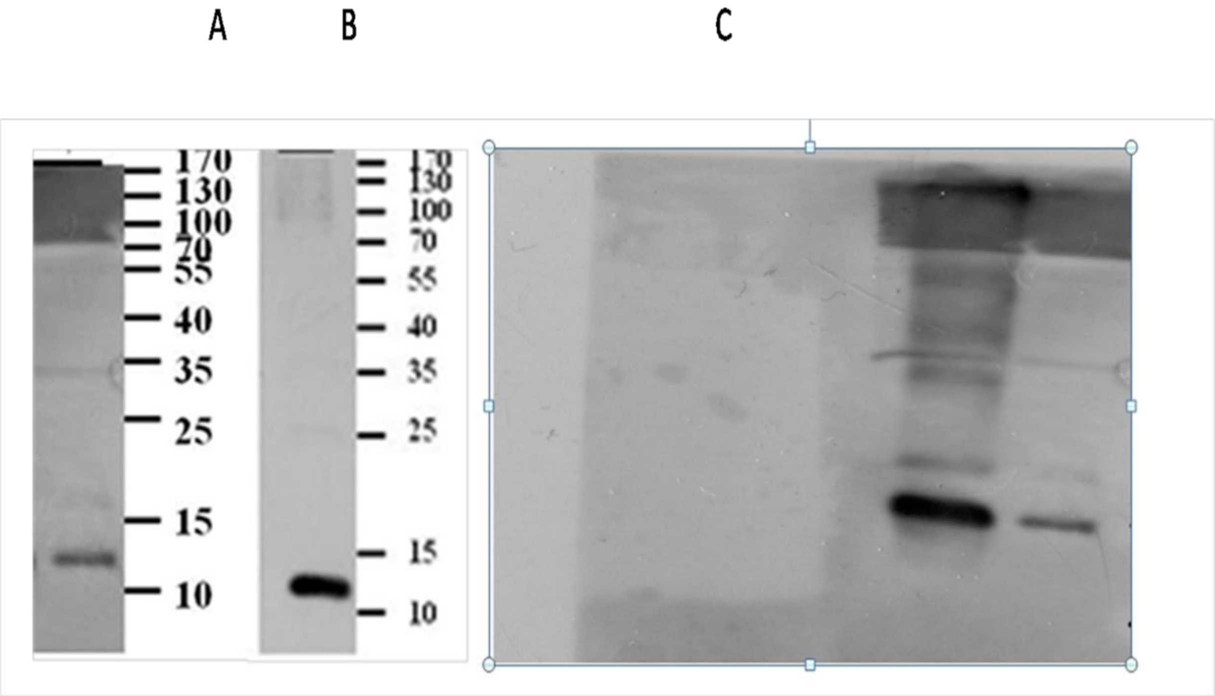

Supplement: Supplementary Information [file srep40749-s1.pdf]
